# *Vox clamantis in deserto*: a survey among Italian psychiatrists on defensive medicine and professional liability

**DOI:** 10.3389/fpsyt.2023.1244101

**Published:** 2023-08-17

**Authors:** Pasquale Scognamiglio, Donato Morena, Nicola Di Fazio, Giuseppe Delogu, Valeria Iniziato, Silvestro La Pia, Pasquale Saviano, Paola Frati, Vittorio Fineschi

**Affiliations:** ^1^Department of Mental Health, ASL Napoli 3 Sud, Torre del Greco, Italy; ^2^Department of Anatomical, Histological, Forensic and Orthopaedic Sciences, Sapienza University of Rome, Rome, Italy; ^3^Department of Mental Health, ASL Napoli 1 Centro, Naples, Italy

**Keywords:** defensive medicine, professional liability, position of guarantee, forensic psychiatry, survey, duty of care, guidelines

## Abstract

Due to recent events, professional liability for psychiatrists in Italy is currently a matter of lively debate. Specifically, overwhelming pressure on psychiatrists’ duties has been brought by regulatory developments, such as the closure of forensic psychiatric hospitals, with the consequent return of offenders to community-based care, and the mental health consequences of the pandemic. According to Italian courts, psychiatrists are not only responsible for diagnostic and therapeutic appropriateness but also for the effects of their interventions on patients, and their behaviors. The aim of this study was to explore the attitude and behaviors of Italian psychiatrists regarding defensive medicine and professional liability. A total sample of 254 psychiatrists was surveyed by means of a quantitative online questionnaire. Most psychiatrists reported practicing defensive medicine (no. 153/254, 60.2%) and felt that their position of guarantee compromised their work in healthcare for patients (no. 138/253, 54.3%). Age correlated inversely with acknowledgment of defensive practices (*r* = −0.245, *p* < 0.001), with younger physicians more prone to defensive medicine (*p* = 0.013), particularly for patients at risk of suicide or violence. Psychiatrists in ‘closed’ settings (hospital wards, residential and rehabilitation centers, mental health service units in prison) reported more malpractice claims (*p* = 0.037) and complaints (*p* = 0.031), as well as a greater propensity to act defensively. In the treatment of patients with violent behavior, suicidal ideation, dual diagnoses, and criminal convictions, defensive practices were associated more with perceived legal risks (*r* = 0.306, *p* < 0.001) than actual legal involvement (*p* > 0.05). Anxiety, anger, and restlessness were common reactions to legal complaints, involving no. 50/254 (19.7%) respondents, with 40% reporting impaired functioning. Most psychiatrists (no. 175/253, 68.9%) were concerned about both civil and criminal laws regarding their professional responsibility, but many were not fully informed about recent legislative regulations and younger physicians resulted scarcely trained in risk management (*p* < 0.001). In conclusion, our findings suggest that defensive medicine is a common phenomenon among psychiatrists and their position of guarantee drives this attitude. Education on legal implications and risk management should be provided starting from the university and continuing over time, to improve the knowledge of young and senior doctors on professional liability and inform their decision-making processes. This would also reduce defensive practices and improve the quality of healthcare. Considering the concerns of younger physicians, as well as of professionals working in acute and high-intensity medical care facilities, there is also an urgent need for a revision of the medical liability to ensure the sustainability of the National Health Service.

## 1. Introduction

The issue of psychiatrists’ professional liability in Italy is currently the subject of a lively debate. This is particularly due to recent regulatory developments, such as the closure of forensic psychiatric hospitals (1), the assignment of patients condemned for offenses to community-based care, and the increased mental health consequences resulting from the pandemic (2).

Medical liability requires that the conduct of a healthcare professional has to be evaluated according to the specialization exercised, in accordance with the nature of the service and the level of danger involved, as well as with attentiveness and adequate professional preparation. Further, the psychiatrist’s responsibility, in addition to reproducing the general medical one, also entails a number of peculiar aspects. This includes taking into account a patient’s mental state and any psychological factors that could affect their treatment, as well as being aware of legal and ethical considerations such as confidentiality and informed consent.

In most countries, physicians’ liability is based upon negligence, a component of medical malpractice. This mainly consists of the sub-standard care provided to patients, i.e., inadequate to what is expected of reasonably competent physicians in their specialistic branch (3).

In civil matters, medical liability for a tort takes the form of compensation for damages resulting from negligent conduct, which entitles the injured party to seek redress. In criminal matters, physicians may be held criminally liable for manslaughters or culpable personal injuries if found to have engaged in negligent, imprudent, or inexperienced behaviors, or for failing to comply with laws, regulations, orders, or professional standards (Article 43 of the Italian Criminal Code).

In addition to maintaining medical responsibility in terms of diagnostic and therapeutic appropriateness, Italian physicians are therefore also responsible for the effects of their interventions on patients and their behaviors, according to the rule of law defined as ‘position of guarantee’ (PoG). PoG refers to a set of protective obligations that doctors have towards their patients, embracing the principle of ‘duty of care’ and the obligation to neutralize all sources of harm (Article 40 of the Criminal Code). For psychiatrists, this kind of vicarious liability is extended to preventing patients from engaging in self-harm or harmful behaviors toward others (4, 5). This obligation should not be confused with the ‘necessity principle’, which provides for intervening by any proportionate means to preserve life and prevent serious injuries (e.g., in psychiatry, with the use of restraint or sedation in case of behaviors at risk for life or serious injuries).

Under PoG, psychiatrists are required to forecast patients’ future behaviors and what their intervention will elicit (6). In recent years, the Italian Supreme Court has frequently held psychiatrists criminally liable for the behaviors of their patients (7).

As an aspect related to medical liability, defensive medicine (DM) refers to all diagnostic and therapeutic procedures performed to avoid the risk of a malpractice liability trial, rather than solely for the benefit of the patient (8).

Defensive practices are traditionally divided into two categories: positive DM, which involves assurance behaviors such as prescribing unnecessary or repetitive tests, procedures, referrals, or additional services; and negative DM, which involves avoidance behaviors such as reluctance to care for high-risk patients or undertake risky procedures (9, 10).

In psychiatry, an example of positive DM would be the case of an outpatient with a risk of violence or suicidal ideation who is hospitalized solely for defensive reasons. An example of negative DM is the reluctance to prescribe or underdose medication to elderly or pregnant women, even if necessary and indicated for their clinical conditions (11). The consequences of DM are negative both for patients, who are exposed to greater risks, and for the healthcare system, due to the significant increase in costs (12). Moreover, physicians themselves are also victims of DM as they are exposed to increasingly stressful working conditions (13). Specializations with high-risk procedures (e.g., surgery), which are intuitively more prone to litigation (14), are the ones in which DM is more likely to be practiced (15).

Psychiatry, with 1% of all paid medical malpractice claims (16, 17), is considered to be among the lower-risk specialties for malpractice claims (15). However, research has shown that DM is frequent among psychiatrists in their routine clinical practice (15). Despite low rates, there are also concerns due to both the steady increase in medical malpractice claims (16) and the potential for criminal and disciplinary actions (18, 19).

North American data show that the most common cause for a medical malpractice claim is patient suicide or attempted suicide, followed by incorrect treatment and breach of confidentiality (16). Other claims relating to medication issues are misdiagnosis, abandonment and unnecessary hospitalization, incorrect or ineffective treatment, improper detainment during hospitalization, boundary violations, and lack of informed consent (18, 20, 21).

In this context, the selection of technical rules and recommendations that guide medical activity in the choice of therapeutic paths is of particular relevance, with an increasingly important role given to clinical risk management.

Several regulatory measures have been implemented over the years to manage clinical risk and patient safety in Italy. In 2008, the Observatory for Good Practices in Patient Safety was established with the objective of disseminating the best examples of patient safety improvement to professionals, citizens, and other stakeholders (22). Health Error Monitoring Information System was established in 2009 to collect data on ‘sentinel events’, which are particularly serious events indicative of a failure in healthcare, and potential future professional liability suits.

In 2017, Law 24/2017, also known as ‘Gelli-Bianco’, introduced significant changes and updated provisions aimed at improving patient safety and healthcare workers’ (HCWs) liability (23). The law emphasizes the importance of risk management for healthcare safety and introduces changes in both civil and criminal liability regimes. In civil matters, Article 7 of the law addresses the responsibility of healthcare facilities, whether private or public, and individual professionals. Healthcare institutions are accountable for the malpractice of HCWs who operate within, as the therapeutic contract is established between the patient and their facilities (24). Conversely, the defense against malpractice claims is entirely up to HCWs who work as independent professionals.

Article 6 of Law 24/2017 addresses criminal liability and introduces changes to the Italian Criminal Code. It states that if death or injuries in healthcare are caused by lack of skill, punishment is to be excluded, provided that the healthcare practitioner acted in compliance with the guidelines published by the National Health System, or, in case of absence, by best healthcare practices.

The guidelines serve as a reference point for physicians in making therapeutic choices and provide a standard for judges assessing the conduct of HCWs. Compliance with the guidelines not only excludes criminal liability but also limits the amount of damages that can be awarded.

Additionally, at the regional level, strengthening of risk management objectives is planned, with the intention of engaging all public and private HCWs in an adequate monitoring, prevention, and risk management function through the following activities: (a) activation of medical audits (systematic analysis of the quality of medical care, including the procedures used for diagnosis and treatment, resource use and process outcomes and patient quality of life), with an analysis of possible activities aimed at securing healthcare paths (25); (b) identification of the risks of inappropriate diagnostic and therapeutic paths and facilitation for the detection of any active or passive defensive medicine activities; (c) preparation and implementation of awareness-raising and continuous training activities to prevent healthcare risks; (d) technical assistance to the facility’s legal department; (e) annual reports on adverse events that have occurred in the facility (26).

Finally, due to concerns about being held criminally responsible for patients’ self-harm or harmful behaviors, there is a risk of misusing regulations regarding the compulsory nature of hospitalizations. In the Italian legal system, these regulations are governed by Law 180/1978, which stipulates that compulsory admission to a general hospital ward is necessary only when the following three elements are present: an urgent intervention is required, necessary treatment is refused, and community-based treatment cannot be implemented effectively.

To our knowledge, there are no studies investigating directly from psychiatrists the extent of the phenomenon of DM and fear of professional liability suits for PoG. However, internationally, some studies have shown that DM is a common practice even among psychiatrists (11, 27). In this field, DM usually takes the form of advice to hospitalize patients with suicidal tendencies even if not necessary, an increase in the frequency of follow-up visits when not warranted, and the prescription of lower doses of drugs than required in the treatment of pregnant women and elderly patients (11).

Regarding the aims of our study, the primary goal was to comprehensively examine the prevalence of DM practices among Italian psychiatrists, considering the influence of both professional liability and PoG. In addition to replicating previous studies in the field, we extended our investigation to include aspects related to the management of patients at risk of violence and psychiatrists’ attitudes towards involuntary hospitalizations. Given the provisions of Law 24/2017, we also included questions regarding psychiatrists’ training on the new regulations and clinical risk management. Lastly, we sought to explore the potential psychological impact of previous malpractice claims on psychiatrists.

## 2. Materials and methods

### 2.1. Study sample

This cross-sectional survey was administered online over a period spanning from 14th March to 28th April 2023. The survey questionnaire was disseminated for telematic self-completion directly to several mental health departments, university clinics, and professional offices. Additionally, social network pages featuring certified psychiatrists and residents in psychiatry were utilized for this purpose. Requests to participate in the survey were distributed throughout the Italian national territory in order to obtain a homogeneous representation of professionals. As such, this was an exploratory study with a targeted sample size of 250 psychiatrists’ respondents aimed at approximating previous reports (11).

As no patients were involved and all data were collected anonymously, there was no need for International Review Board approval.

### 2.2. Survey instrument

Before completing the questionnaire, a brief explanation of the survey’s purpose and the methods of data collection, management, and communication was provided to the participants. They were also informed that the data collected would be handled in an aggregated form to ensure their anonymity. Participants were required to provide informed consent before accessing the questionnaire. Additionally, the survey included questions about personal experiences related to malpractice claims, disciplinary investigations, and exposure to medico-legal literature about DM. Demographic data (gender, age, education/qualification, seniority, usual place of employment, department position) were also collected.

The main questionnaire was derived from the 13-item questionnaire developed by Reuveni et al. (11), which was itself a replication of a questionnaire created by Passmore and Leung (27). In addition to the four defensive practice domains of the Reuveni et al. questionnaire, which include treating suicidal patients (1), treating pregnant women (2), initiating or changing drug treatment (3), and treating elderly patients (4), we added questions about treating violent patients (5). As part of the survey on perceptions of DM (6), we also added further questions regarding the impact of PoG on the therapeutic relationship and on choices regarding involuntary hospitalization. Questions were proposed regarding Law 24/2017 – ‘Gelli-Bianco’, clinical risk management, and the training received in this field (7) in light of recent regulatory developments. The section concerning the ‘Gelli-Bianco’ Law consisted of multiple-choice questions, whereas the items related to the other dimensions were scored on a Likert scale ranging from 1 (with no patient) to 5 (with every patient) (e.g., ‘Do you recommend psychiatric hospitalization for patients who reports violent intentions even if not justified by their mental state?’).

Furthermore, if the interviewees answered affirmatively to the question regarding involvement in a past criminal complaint or claim for compensation by a patient or their family member, as for Reuveni et al.’s questionnaire, they were asked about their feelings (anxious, restless, angry, loss of energy or tired, guilty and mistrustful) and functioning (sleep problems and interference with work, family or social activities) during the period of involvement.

The questionnaire was edited and distributed using the free online tool “Google Forms” (Google LLC, Mountain View, California, CA, United States).

### 2.3. Data and statistical methods

Descriptive statistics were used to examine physicians’ characteristics, their opinions about DM, and their experience with clinical risk management. For those who had responded positively to involvement in a past criminal complaint or claim for compensation, a descriptive analysis was performed to highlight the consequences on their psychological and functional status at the time of the claim.

T-test for independent samples was used to analyze the differences between physicians who worked in ‘open’ services (community mental health centers, addiction service units, private practices) – i.e., where patients and other people could access on a voluntary basis and for a brief time – and in ‘closed’ services (hospital wards, residential and rehabilitation centers, mental health service units in prison) – i.e., where inpatients are entrusted to healthcare personnel, in some cases even compulsorily.

Three groups of participants were formed according to aggregate age: ‘<35’, ‘36–50’, and ‘>50’. Differences between these three groups of participants in relation to items of the questionnaire, risk management education, and ‘Gelli-Bianco’ Law knowledge/reading were investigated with a One-Way Analysis of Variance (ANOVA). For pairwise comparisons, Games-Howell post-hoc tests were used due to the different sample sizes within each group. Levene’s test was performed to verify whether the variances of the three groups were significantly different. If Levene’s test was significant (*p* < 0.05), Welch’s *F* was considered.

Pearson correlation coefficients were calculated for acknowledgment of defensive practice and the 26 items about defensive behaviors, age, seniority, and involvement in complaints. As proposed by Reuveni et al. (11), acknowledgement of practicing DM was based on the direct question: ‘Do you practice defensive medicine?’. Admitting to practicing DM with at least half of the patients was considered a valid cut-off.

Finally, to assess the internal reliability of our questionnaire, Cronbach’s *α* of the 26 items about defensive behaviors was calculated, resulting in good internal consistency: *α* = 0.82. Reported *p* values are two-sided. All analyses were performed using IBM SPSS V.25.0 statistical software.

## 3. Results

A summary of findings can be found in Supplementary Table S1.

### 3.1. Sample characteristics

Table 1 summarizes the demographic characteristics of the study sample. The final respondent sample consisted of 254 psychiatrists, no. 45 (17.7%) under the age of 35, no. 135 (53.1%) between 36 and 50 years and no. 73 (28.7%) were over 50. Approximately 60% of the sample was composed of women, and the vast majority of the sample consisted of certified psychiatrists (90.9%). There was a strong representativity by seniority range (almost were consultants, no. 191, 75.2%). The division into two categories based on workplace, namely the ‘open’ group (no. 175, 68.9%) and the ‘closed’ group (no. 78, 30.7%), provided a sufficient representation despite the imbalance in workplace distribution.

**Table 1:** Socio-demographic characteristics of subjects of the sample of Italian psychiatrists.

		No.	%
Gender	Male	100	39.4
	Female	151	59.4
	Other	1	0.4
Age	<35	45	17.7
	36–50	135	53.1
	>50	73	28.7
Educational Qualification	Specialist	231	90.9
	Resident	19	7.5
Seniority (after specialization)	<10	124	48.8
	11–20	66	26.0
	>20	63	24.8
Usual place of work	Hospital ward (SPDC)	56	22.0
	Community mental health center (CSM or CPS)	151	59.4
	Residential and rehabilitation center	12	4.7
	Mental health service unit in prison	10	3.9
	Addiction service unit (Ser.D.)	11	4.3
	Private practice	13	5.1
Department position	Resident	20	7.9
	Consultant	191	75.2
	Department director	23	9.1
	Free professional	13	5.1
	Researcher	1	4
	Professor	3	1.2

### 3.2. Defensive practice

The full list of questions and answers obtained from our 26-item questionnaire is reported in Table 2. Prior to the completion of the questionnaire, information regarding involvement in professional liability cases was requested. Over half of the interviewees (no. 132, 52.0%) reported exposure to the risk of medical negligence that could have led to a malpractice claim, though only no. 50 (19.7%) affirmed to have effectively received a complaint.

**Table 2:** Full list of questions and answers obtained from the survey.

Questions	Total Answers	Answers
The following questions refer to the history of criminal complaints and/or compensation claims		
1. Were you ever involved in a case of medical negligence that could have led to a malpractice claim? (CriminalComplaints1)	254/254	Yes no. 132 (52.0%)
		No no. 122 (48.0%)
2. Have you ever been involved in an “internal investigation” or been summoned to a departmental disciplinary board? (CriminalComplaints2)	254/254	Yes no. 41 (16.1%)
		No no. 213 (83.9%)
3. Do you regularly read medico-legal literature and keep yourself updated on the scope of medical liability? (CriminalComplaints3)	254/254	Yes no. 155 (61.0%)
		No no. 99 (39.0%)
4. If yes (to the previous question), to what extent do these issues affect your decision-making in daily clinical practice? (CriminalComplaints4)	191/254	Not at all no. 11 (4.3%)
		Slightly no. 96 (37.8%)
		To a certain extent no. 69 (27.2%)
		Very Much no. 15 (5.9%)
The following questions refer to the treatment of suicidal patients		
1. Do you advise psychiatric hospitalization to a suicidal patient even if not warranted according to his/her mental state?	254/254	With no patient no. 13 (5.1%)
(SuicidalPatients1)		In a few patients no. 103 (40.6%)
		With half of the patients no. 30 (11.8%)
		With most patients no. 91 (35.8%)
		With every patient no. 17 (6.7%)
2. Do you increase follow-up of a suicidal patient even if it is not warranted according to his/her mental state?	254/254	With no patient no. 2 (0.8%)
(SuicidalPatients2)		In a few patients no. 38 (15.0%)
		With half of the patients no. 17 (6.7%)
		With most patients no. 123 (48.4%)
		With every patient no. 74 (29.1%)
3. Do you usually initiate contact with a family member or other support networks for a patient reporting suicidal intentions even if not justified by his/her mental status?	254/254	With no patient no. 5 (2.0%)
(SuicidalPatients3)		In a few patients no. 38 (15.0%)
		With half of the patients no. 27 (10.6%)
		With most patients no. 127 (50.0%)
		With every patient no. 57 (22.4%)
4. Do you consult with a senior psychiatrist / head of the service or of the department?	254/254	With no patient no. 9 (3.5%)
(SuicidalPatients4)		In a few patients no. 111 (43.7%)
		With half of the patients no. 42 (16.5%)
		With most patients no. 67 (26.4%)
		With every patient no. 25 (9.8%)
5. Do you usually refer these patients to another professional (psychologist / social worker)?	252/254	With no patient no. 24 (9.4%)
(SuicidalPatients5)		In a few patients no. 96 (37.8%)
		With half of the patients no. 52 (20.5%)
		With most patients no. 70 (27.6%)
		With every patient no. 10 (3.9%)
6. Do you prescribe drugs to a patient reporting suicidal intentions even if not justified by his/her mental status?	252/254	With no patient no. 27 (10.6%)
(SuicidalPatients6)		In a few patients no. 104 (40.9%)
		With half of the patients no. 49 (19.3%)
		With most patients no. 63 (24.8%)
		With every patient no. 9 (3.5%)
The following questions refer to the management of violent patients		
1. Do you advise psychiatric hospitalization to a patient reporting violent intentions even if not warranted according to his/her mental state?	253/254	With no patient no. 35 (13.8%)
(ViolentPatients1)		In a few patients no. 131 (51.6%)
		With half of the patients no. 47 (18.5%)
		With most patients no. 33 (13.0%)
		With every patient no. 7 (2.8%)
2. Do you increase follow-up of a patient reporting violent intentions even if not warranted according to his/her mental state?	253/254	With no patient no. 12 (4.7%)
(ViolentPatients2)		In a few patients no. 81 (31.9%)
		With half of the patients no. 48 (18.9%)
		With most patients no. 85 (33.5%)
		With every patient no. 27 (10.6%)
3. Do you usually initiate contact with a family member or other support networks for a patient reporting violent intentions even if not justified by his/her mental status?	253/254	With no patient no. 8 (3.1%)
(ViolentPatients3)		In a few patients no. 52 (20.5%)
		With half of the patients no. 50 (19.7%)
		With most patients no. 100 (39.4%)
		With every patient no. 43 (16.9%)
4. Do you consult with a senior psychiatrist / head of the service or of the department?	254/254	With no patient no. 11 (4.3%)
(ViolentPatients4)		In a few patients no. 85 (33.5%)
		With half of the patients no. 45 (17.7%)
		With most patients no. 89 (35.0%)
		With every patient no. 24 (9.4%)
5. Do you usually refer these patients to another professional (psychologist / social worker)?	250/254	With no patient no. 35 (13.8%)
(ViolentPatients5)		In a few patients no. 106 (41.7%)
		With half of the patients no. 53 (20.9%)
		With most patients no. 46 (18.1%)
		With every patient no. 10 (3.9%)
6. Do you prescribe drugs to a patient reporting violent intentions even if not justified by his/her mental status?	253/254	With no patient no. 14 (5.5%)
(ViolentPatients6)		In a few patients no. 74 (29.1%)
		With half of the patients no. 50 (19.7%)
		With most patients no.94 (37.0%)
		With every patient no. 21 (8.3%)
The following questions refer to initiating or changing patients’ medications		
1. When initiating a new medication do you inform your patient about severe yet rare side effects?	254/254	With no patient no. 9 (3.5%)
(PatientsMedication1)		In a few patients no. 54 (21.3%)
		With half of the patients no. 23 (9.1%)
		With most patients no. 77 (30.3%)
		With every patient no. 91 (35.8%)
2. Do you record having explained to the patient about the side effects before initiating new medication?	254/254	With no patient no. 66 (26.0%)
(PatientsMedication2)		In a few patients no. 91 (35.8%)
		With half of the patients no. 11 (4.3%)
		With most patients no. 39 (15.4%)
		With every patient no. 47 (18.5%)
3. Do you inform your patient about the increased risk of suicidality after initiating treatment with SSRIs (as stated by AIFA warning)?	253/254	With no patient no. 87 (34.3%)
(PatientsMedication 3)		In a few patients no. 87 (34.3%)
		With half of the patients no. 18 (7.1%)
		With most patients no. 32 (12.6%)
		With every patient no. 29 (11.4%)
The following questions refer to the treatment of pregnant women		
1. Do you avoid prescribing medication for pregnant women altogether?	253/254	With no patient no. 25 (9.8%)
(Pregnant1)		In a few patients no. 85 (33.5%)
		With half of the patients no. 37 (14.6%)
		With most patients no. 85 (33.5%)
		With every patient no. 21 (8.3%)
2. If you decide to prescribe a new drug for a pregnant patient, do you obtain informed consent other than the standard model?	253/254	With no patient no. 62 (24.4%)
(Pregnant2)		In a few patients no. 36 (14.2%)
		With half of the patients no. 6 (2.4%)
		With most patients no. 34 (13.4%)
		With every patient no. 115 (45.3%)
3. If you decide to initiate new medication for a pregnant patient do you prescribe smaller dosages than customary?	253/254	With no patient no. 32 (12.6%)
(Pregnant3)		In a few patients no. 45 (17.7%)
		With half of the patients no. 17 (6.7%)
		With most patients no. 91 (35.8%)
		With every patient no. 68 (26.8%)
The following questions refer to the treatment of elderly patients		
1. When you initiate antipsychotic treatment in an elderly patient do you inform the patient or his/her family member about increased risk of cerebrovascular events?	253/254	With no patient no. 33 (13.0%)
(Elderly1)		In a few patients no. 84 (33.1%)
		With half of the patients no. 25 (9.8%)
		With most patients no. 48 (18.9%)
		With every patient no. 63 (24.8%)
2. If you decide to prescribe a new medication to an elderly patient do you prescribe smaller dosages than customary?	253/254	With no patient no. 3 (1.2%)
(Elderly2)		In a few patients no. 15 (5.9%)
		With half of the patients no. 13 (5.1%)
		With most patients no. 117 (46.1%)
		With every patient no. 105 (41.3%)
The following questions refer to the practice of Defensive Medicine		
1. Generally, do you practice defensive medicine in your daily practice?	254/254	With no patient no. 15 (5.9%)
(Defensive Medicine1)		In a few patients no. 86 (33.9%)
		With half of the patients no. 70 (27.6%)
		With most patients no. 59 (23.2%)
		With every patient no. 24 (9.4%)
2. Do you think your position of guarantee compromises the relationship with some patients (users with violent behavior, suicidal ideation, dual diagnosis, pending charges)?	253/254	With no patient no. 26 (10.2%)
(Defensive Medicine2)		In a few patients no. 90 (35.4%)
		With half of the patients no. 50 (19.7%)
		With most patients no. 63 (24.8%)
		With every patient no. 24 (9.4%)
3. Do you think your position of guarantee compromises negatively impacts the goal of care with some patients (users with violent behavior, suicidal ideation, dual diagnosis, pending charges)?	253/254	With no patient no. 33 (13.0%)
(Defensive Medicine3)		In a few patients no. 82 (32.3%)
		With half of the patients no. 50 (19.7%)
		With most patients no. 64 (25.2%)
		With every patient no. 24 (9.4%)
4. Have you ever prescribed or changed the dosage of a drug thinking primarily of your position of guarantee and only subordinate to the user’s clinical conditions?	253/254	With no patient no. 46 (18.1%)
(Defensive Medicine4)		In a few patients no. 116 (45.7%)
		With half of the patients no. 54 (21.3%)
		With most patients no. 29 (11.4%)
		With every patient no. 8 (3.1%)
5. Have you ever required compulsory psychiatric treatment for a patient, mostly thinking about your position of guarantee rather than about the user’s clinical conditions?	252/254	With no patient no. 96 (37.8%)
(Defensive Medicine5)		In a few patients no. 110 (43.3%)
		With half of the patients no. 27 (10.6%)
		With most patients no. 16 (6.3%)
		With every patient no. 3 (1.2%)
6. Have you ever required compulsory psychiatric treatment for a patient mostly on the basis of external solicitations (e.g., the police, mayor, associations, etc.)?	253/254	With no patient no. 100 (39.4%)
(Defensive Medicine6)		In a few patients no. 123 (48.4%)
		With half of the patients no. 21 (8.3%)
		With most patients no. 7 (2.8%)
		With every patient no. 2 (0.8%)
The following questions refer to the Italian Legislation and to Law 24/2017, the so-called “Gelli-Bianco”		
1. For which legal matter do you believe you are most at legal risk for your profession?	253/254	Civil Law no. 29 (11.4%)
(GelliBianco1)		Criminal Law no. 49 (19.3%)
		Both no. 175 (68.9%)
2. Have you read the content of Law 24/2017, the so-called “Gelli-Bianco”?	253/254	Yes no. 93 (36.6%)
(GelliBianco2)		No no. 73 (28.7%)
		Partly no. 87 (34.3%)
3. If yes (to the previous question), do you believe that Law 24/2017, the so-called “Gelli-Bianco”, made appropriate corrections to the previous regulations in terms of greater guarantees for psychiatrists?	177/254	Yes no. 8 (3.1%)
(GelliBianco3)		No no. 63 (24.8%)
		Partly no. 106 (41.7%)
4. If yes (to the previous question), for which legal matter do you believe that Law 24/2017, the so-called “Gelli-Bianco”, has provided greater guarantees for psychiatrists?	169/254	Civil Law no. 58 (22.8%)
(GelliBianco4)		Criminal Law no. 43 (16.9%)
		Both no. 68 (26.8%)
5. Have you ever participated in training events on clinical risk?	254/254	Yes no. 152 (59.8%)
(GelliBianco5)		No no. 102 (40.2%)
6. If yes (to the previous question), at which kind of event did you participate?	157/254	Master’s topic no. 12 (7.6%)
(GelliBianco6)		Specific master no. 6 (3.8%)
		Online course no. 56 (35.7%)
		Subject of the degree course no. 18 (11.5%)
		In departmental course no. 70 (44.6%)
		Not departmental course no. 54 (34.4%)
7. Do you think you have received adequate training regarding risk prevention and patient safety?	249/254	Yes no. 27 (10.6%)
(GelliBianco7)		No no. 127 (50.0%)
		Partly no. 95 (37.4%)
8. Do you think that clinical risk management can contribute to reducing complaints for professional liability?	252/254	Yes no. 170 (67.5%)
(GelliBianco8)		No no. 21 (8.3%)
		Does not know no. 61 (24.2%)
9. Usually, you follow the Guidelines and Good Clinical Practices	252/254	- Exclusively as based on evidence and therefore of proven clinical efficacy no. 45 (17.9%)
(GelliBianco9)		- Both as evidence-based and therefore of proven clinical efficacy, and to reduce the risk of losing in the event of complaints for professional liability no. 177(70.2%)
		- Mostly to reduce the risk of losing in the event of complaints for professional liability no. 30 (11.9%)

A good number of the doctors, no. 155 (CriminalComplaints3 61.0%), kept a constant update on medical-legal and medical liability literature, although the majority of respondents were not influenced in clinical practice (CriminalComplaints4 ‘Not at all’ no. 11, 4.3%, and ‘Slightly’ no. 96, 37.8%). Based on the responses given between ‘With half the patients’ and ‘With every patient’ (‘≥50% group’), more than half of the psychiatrists adopted a defensive attitude with suicidal patients, despite a balance in terms of medication prescription.

For violent patients, there was a lower tendency to hospitalization (‘With no patient’ no. 35, 13.8%, and ‘In a few patients’ no. 131, 51.6%) as well as refer these patients to another professional (‘With no patient’ no. 35, 13.8%, and ‘In a few patients’ no. 106, 41.7%) while the frequency of defensive behaviors was high for the additional items.

For medical prescriptions there was a tendency to inform patients about severe yet rare side effects (PatientsMedication1 ‘≥50% group’ no. 191, 75.2%), while the percentage of those who recorded this information was lower (PatientsMedication2 ‘≥50% group’ no. 97, 38.2%), as well as providing information on the suicidal risk associated with SSRIs (PatientsMedication3 ‘≥50% group’ no. 79, 31.1%).

The percentages of non-prescription drugs in pregnant women were high (Pregnant1 ‘≥50% group’, no. 143, 56.4%), as was the use of specific informed consent collection models (Pregnant2 ‘≥50% group’, no. 155, 61.1%) and of lower dosages than the customary (Pregnant3 ‘≥50% group’, no. 176, 69.3%). The latter attitude was also applied to elderly patients (Elderly2 ‘≥50% group’, no. 235, 92.5%), while the information on cerebrovascular events was more balanced.

Based on the specific questions about DM, it was determined that the majority of respondents were aware of the practice of DM (Defensive Medicine1, no. 153, 60.2%), as were of the high implications of PoG on the therapeutic relationship (Defensive Medicine2 ‘≥50% group’, no. 137, 53.9%) and the goal of care (Defensive Medicine3 ‘≥50% group’, no. 138, 54.3%), specifically in relation to individuals with violent behavior, suicidal ideation, dual diagnosis, and pending charges. PoG had a lesser impact on psychiatrists’ decisions in terms of pharmacological prescriptions (Defensive Medicine4 ‘≥50% group’, no. 91, 35.8%) and compulsory psychiatric treatment (Defensive Medicine5 ‘≥50% group’, no. 142, 18.1%), while external factors had little influence on the decision to hospitalize (Defensive Medicine6 ‘≥50% group’, no. 30, 11.9%).

### 3.3. Italian legislation and on Law 24/2017, so-called ‘Gelli-Bianco’

Regarding the current legislation, concerns were expressed regarding both civil and criminal aspects of professional liability (GelliBianco1 no. 175, 68.9%). However, a fair number of doctors had not read the contents of Law 24/2017 (GelliBianco2 no. 73%, 28.7%) or had only partially read it (GelliBianco2 no. 87, 34.3%). Only a small part of respondents affirmed the improved protections for psychiatrists provided by Law 24/2017 (GelliBianco3 no. 8%, 3.1%), while opinions regarding the effective field of application of the law were very heterogeneous.

### 3.4. Risk management

The percentage of psychiatrists who had participated in risk management training was only slightly more than half (GelliBianco5 no. 152, 59.8%). Most of the training was performed in departmental (no. 70, 44.6%) and non-departmental (no. 54, 34.4%) courses but also the use of digital platforms was well represented (no. 56, 35.7%). Further analyses show no differences in risk management training between psychiatrists in different workplaces (e.g., ‘open’ and ‘closed’ groups *p* = 0.902), while greater participation was recorded by older groups (Supplementary Table S4).

### 3.5. Feelings and functioning of physicians involved in legal proceedings for professional liability

Out of 254 participants, no. 50 (19.7%) reported being involved in a past civil or criminal complaint by one of their patients or their family members. Considering the sum of those who responded ‘To a certain extent’ or ‘Very Much’ (CE-VM), the most frequent emotion reported was anxiety (‘To a certain extent’ no. 21, 42%, ‘Very Much’ no. 22, 44%, CE-VM = 86%), followed by anger (‘To a certain extent’ no. 13, 26%, ‘Very Much’ no. 28, 56%, CE-VM = 82%), restlessness (‘To a certain extent’ no. 22, 44%, ‘Very Much’ no. 9, 18%, CE-VM = 62%), loss of energy/fatigue (‘To a certain extent’ no. 25, 50%, ‘Very Much’ no. 4, 8%, CE-VM = 58%), distrustful (‘To a certain extent’ no. 20, 40%, ‘Very Much’ no. 9, 18%, CE-VM = 58%), and sleeping problems (‘To a certain extent’ no. 18, 36%, ‘Very Much’ no. 9, 18%, CE-VM = 54%). Impairment in functioning at work, in family relations, or in social activities affected just under half of the sample (‘To a certain extent’ no. 16, 32.7, ‘Very Much’ no. 4, 8.2%, CE-VM = 40.9%), while the least represented emotion, reported by less than one-fifth of the sample, was guilt (‘To a certain extent’ no. 7, 14%, ‘Very Much’ no. 2, 4%, CE-VM = 18%).

### 3.6. T-test results for ‘open’–‘closed’ groups

In the comparison of ‘open’ and ‘closed’ groups (Supplementary Table S3), it was found that psychiatrists who worked in closed environments were more likely to be involved in malpractice claims (CriminalComplaints1 *p* = 0.037) and complaints (Involvement in complaints *p* = 0.031).

In the management of suicidal individuals, the ‘closed’ group tended to hospitalize more (SuicidalPatients1 *p* = 0.015), have more contact with family or other support networks (SuicidalPatients3 *p* < 0.001), and consult an experienced psychiatrist (SuicidalPatients4 *p* = 0.024).

In the management of individuals at risk of violence, the ‘closed’ group tended to have more contact with family or other support networks (ViolentPatients3 *p* = 0.016), consult an experienced psychiatrist (ViolentPatients4 *p* = 0.015), and refer the person to another professional (ViolentPatients5 *p* = 0.029).

There was a tendency among the ‘open’ group to be more reluctant to prescribe drugs to pregnant women (Pregnant1 *p* = 0.048).

### 3.7. ANOVA

Groups with different ages showed significant differences (Supplementary Table S4). The group with younger people was less involved in medical liability (CriminalComplaints1 *p* = 0.015) and in cases of complaints and/or claims for compensation (Involvement in complaints *p* = 0.005) compared to the other two groups. Instead, there was also a correlation between age and participation in an internal investigation or in a departmental disciplinary board (CriminalComplaints2 *p* < 0.001).

In the management of suicidal individuals, the ‘<35 group’ consulted senior psychiatrists more frequently than the other two groups (SuicidalPatients4 *p* < 0.001) and were more likely to refer them to other professionals than the ‘>50 group’ (SuicidalPatients5 *p* = 0.036).

In the management of individuals at risk of violence, the three groups consulted senior psychiatrists more frequently in an inverse proportion to their age (ViolentPatients4 *p* < 0.001). The ‘<35 group’ was more likely to refer patients to other professionals (ViolentPatients5 *p* = 0.038) and to prescribe medication even when not justified by the psychopathological conditions, both compared to the ‘36–50’ and the ‘>50’ groups (ViolentPatients6 *p* = 0.010).

Significant differences between groups also emerged regarding pharmacotherapy prescription, where the ‘>50 group’ showed an increased tendency to DM, with greater attention to informing patients about serious but rare side effects (PatientsMedication1 *p* < 0.001), as well as the increased risk of suicide associated with the use of SSRIs (PatientsMedication3 *p* = 0.003) and cerebrovascular events in elderly patients (Elderly1 *p* < 0.001). However, both the ‘<35’ and ‘36–50’ groups (Acknowledgement of defensive practice *p* = 0.013) reported practicing more DM than the ‘>50 group’, while there was no significant difference between the ‘<35’ and ‘36–50’ groups. Participation in risk management training was proportional to age (GelliBianco5 *p* < 0.001).

### 3.8. Correlations

The analysis of the correlations (Table 3) showed that the awareness of practicing defensive procedures correlated negatively with information in the medico-legal and medical liability fields (CriminalComplaints3 *r* = −0.126, *p* = 0.045) and positively with the awareness that these insights influence the decision-making process in daily clinical practice (CriminalComplaints4 *r* = 0.189, *p* = 0.009).

**Table 3:** Defensive medicine: frequencies and correlations with acknowledgment of defensive practice.

	Correlation with acknowledgement of defensive practice
*Criminal Complaints*	
Being involved in a malpractice case (CriminalComplaints1)	−0.056
Being involved in an internal inquiry and/or convocated by a disciplinary board (CriminalComplaints2)	0.037
Education on medical liability (CriminalComplaints3)	−0.126*
Influence of education on medical liability in clinical practice (CriminalComplaints4)	0.189**
*Suicidal Patients*	
Advises unwarranted hospitalisation (SuicidalPatients1)	0.213**
Increases follow-up (SuicidalPatients2)	0.216**
Initiates contact with family (SuicidalPatients3)	0.163**
Consults senior psychiatrist (SuicidalPatients4)	0.270**
Refers to another professional (SuicidalPatients5)	0.203**
Prescribes medication without indication (SuicidalPatients6)	0.295**
*Violent Patients*	
Advises unwarranted hospitalisation (ViolentPatients1)	0.276**
Increases follow-up (ViolentPatients2)	0.184**
Initiates contact with family (ViolentPatients3)	0.079
Consults senior psychiatrist (ViolentPatients4)	0.291**
Refers to another professional (ViolentPatients5)	0.188**
Prescribes medication without indication (ViolentPatients6)	0.262**
*Patients Medication*	
Informs about severe yet rare side effects (PatientsMedication1)	−0.069
Records that explained about side effects (PatientsMedication2)	−0.086
Informs of increased risk of suicidality (PatientsMedication3)	−0.117
*Pregnant patients*	
Avoids medication altogether (Pregnant1)	0.060
Collects different consent (Pregnant2)	0.081
Prescribes a smaller dosage (Pregnant3)	0.227**
*Elderly patients*	
Informs of cerebrovascular diseases risk (Elderly1)	−0.111
Prescribes a smaller dosage (Elderly2)	0.193**
*Defensive Medicine*	
Admission of practising defensive medicine (DefensiveMedicine1)	0.831**
Believe the position of guarantee influences physicians’ relationships with certain types of patients, e.g., those with violent behavior, suicidal ideation, dual diagnoses, criminal convictions (DefensiveMedicine2)	0.349**
Believe the position of guarantee adversely affects the clinical outcome of certain types of patients, e.g., those with violent behavior, suicidal ideation, dual diagnoses, criminal convictions (DefensiveMedicine3)	0.306**
Prioritize the position of guarantee over patient needs in prescribing drugs (DefensiveMedicine4)	0.350**
Prioritize the position of guarantee over patient needs in involuntary hospitalization (DefensiveMedicine5)	0.346**
Involuntarily hospitalization due to external pressure (rather than a medical need) (DefensiveMedicine6)	0.267**
*Demographic*	
Age (years)	−0.245**
Age by groups*	−0.178**
Seniority (years)	−0.247**
*Others*	
Involvement in complaints	−0.010
Acknowledgement of defensive practice	1

*p < 0.05 and **p < 0.001. Bold values denote statistical significance at the *p* < 0.05 level.

In the treatment of individuals with suicidal tendencies, all elements correlated positively with the assessment of the use of defensive procedures in treatment: hospitalization (SuicidalPatients1 *r* = 0.213, *p* = 0.001), increase in follow-up (SuicidalPatients2 *r* = 0.216, *p* = 0.001), contacts with family members or other support networks (SuicidalPatients3 *r* = 0.163, *p* = 0.009), consulting with a senior psychiatrist (SuicidalPatients4 *r* = 0.270, *p* < 0.001), referring the person to another professional (SuicidalPatients5 *r* = 0.203, *p* = 0.001), prescribing new medications (SuicidalPatients6 *r* = 0.295, *p* < 0.001).

In the treatment of individuals at risk of violence, the same positive correlations were found as in suicidal people, with the exception of initiating contact with family members or other support networks (ViolentPatients3 *r* = 0.079, *p* = 0.211).

Regarding medication prescriptions, informing patients about serious but rare side effects, about the increased suicidal risk associated with SSRIs, as well as the note in the medical record of having given information about possible side effects did not correlate with the perception of practicing MD. Conversely, prescribing lower dosages than usual for pregnant women (Pregnant3 *r* = 0.227, *p* < 0.001) and elderly (Elderly2 *r* = 0.193, *p* = 0.002) was associated with a positive correlation.

Significant correlations also emerged about the consideration of the PoG. Specifically, with the perception that it compromises the relationship with peculiar patients (i.e., individuals with violent behavior, suicidal ideation, dual diagnosis, pending charges) (DefensiveMedicine2 *r* = 0.349, *p* < 0.001); that has a negative impact on the goal of care (DefensiveMedicine3 *r* = 0.306, *p* < 0.001); that is of priority importance compared to the clinical conditions of the patients for the prescription of drugs (DefensiveMedicine4 *r* = 0.350, *p* < 0.001), and for the decision on involuntary hospitalization (DefensiveMedicine5 *r* = 0.346, *p* < 0.001). Implementing an involuntary hospitalization primarily on the basis of solicitations external to the health care system (e.g., Police, Mayor, Associations, etc.) also correlated with a greater perception of practicing DM (DefensiveMedicine6, *r* = 0.267, *p* < 0.001).

According to demographic analysis, the perception of practicing DM was negatively correlated with age (*r* = −0.245, *p* < 0.001) and seniority (*r* = −0.247, *p* < 0.001).

Involvement in medical liability cases, disciplinary proceedings, or complaints did not significantly correlate with perceptions of practicing DM.

## 4. Discussion

In this study, we aimed to expand our understanding of the prevalence of DM among psychiatrists in Italy by exploring aspects that have not been investigated in previous studies conducted in other countries. Specifically, we investigated (1) the impact of DM on clinical practice, (2) the effect of PoG on the therapeutic relationship, and (3) on decision-making related to involuntary hospitalization in real-world settings. This was particularly important given the unique context of Italy’s mental health services system. In the 70s, Italy has undergone significant reforms, resulting in the closure of asylums and the transfer of individuals with mental health problems to community-based healthcare. In recent years, this model has applied also to patients who committed crimes. Italian mental health services are primarily provided through community facilities, where patients receive outpatient care. Referrals are made for hospitalizations or residential therapeutic-rehabilitation programs as needed. Additionally, there are centers for pathological addictions more or less integrated into mental health departments depending on regional laws, particularly for patients with dual diagnoses.

To obtain a comprehensive understanding of DM practices among psychiatrists in Italy, we endeavored to engage professionals working in various service settings such as community mental health and/or addiction centers, hospitals, and rehabilitation facilities. Our study aimed to investigate potential differences in DM practices among psychiatrists depending on their work setting. Additionally, we aimed to explore the factors that contribute to DM practices, such as fear of litigation, criticalities in the medical-patient alliance, and pressure from other organizations. By analyzing these factors, we sought to provide insights into how DM can be minimized and how mental health services can be improved.

Anyway, the main results of the study were that (1) most psychiatrists reported practicing DM, and felt it compromised care; (2) younger doctors more often reported liability concerns and acknowledged defensive practice; (3) psychiatrists in ‘closed’ settings more often reported legal issues and took more defensive actions; (4) most psychiatrists were concerned about both civil and criminal laws regarding their professional responsibility, but many were not fully informed about recent legislative regulations; (5) defensive practice was linked more to perceiving legal risks as impacting care than actual legal involvement; (6) anxiety, anger, and restlessness were common reactions to legal complaints, with slightly less than half reporting impaired functioning.

In general terms, it emerged that DM is a common phenomenon among Italian psychiatrists, with over half of the interviewed sample (no. 153/254, 60.2%) reporting being aware of practicing it. DM had implications in almost all the clinical situations investigated, i.e., those involving suicidal and violent patients, pregnant women, and the elderly, albeit with some exceptions (e.g., it was infrequent to provide information on suicidal risks associated with SSRIs).

The condition of PoG was a significant concern, as evidenced by the high percentage of affirmative responses regarding its impact on the therapeutic relationship and the treatment goals for some individuals with the most complex care needs (such as those with violent behavior, suicidal ideation, dual diagnosis, or pending charges). Defensive measures can result in unnecessary tests, procedures, and costs. These findings have crucial implications for the mental health field, as DM can lead not only to increased costs but also to unwarranted coercive measures or the use of unnecessary medication. Additionally, due to fear of their PoG, healthcare professionals may prioritize legal protection over the care and well-being of their patients.

However, it is important to note that the use of defensive procedures may not always be detrimental to patient care, and in some cases, it may be necessary to ensure the safety of the patient.

An example is information on the side effects and risks associated with the use of medications (e.g., SSRIs and antipsychotics), the attention to dosage for elderly patients or pregnant women, as well as recording of the information provided.

Healthcare professionals should be aware of the potential risks and benefits of DM and should prioritize patient care over legal protection when making treatment decisions. That’s why PoG is a great limitation to offering better treatments to patients. An example is the hospitalization of patients that, especially when involuntary, could be determined by several non-clinical conditions, including pressures from family members or police (28).

On this point, a significant finding was that almost all respondents, despite concerns about their own PoG, adhere completely to their care duties. As a matter of fact, involuntary hospitalizations for reasons of self-protection were infrequent, and those due to external pressures even rarer.

Although the incidence of malpractice claims for psychiatrists is estimated at just under 3% per year (15), more than half of the interviewees reported involvement in a case of medical negligence that could have led to a malpractice claim.

Moreover, in about a fifth of the interviewees (no. 50/254, 19.68), such risks resulted in civil or criminal proceedings. These frequencies were similar to those found in the study by Reuveni et al. (11), as were the outcomes of these situations. In both studies, in fact, the most represented feelings among the emotions experienced by the involved psychiatrists were anxiety, anger, and restlessness. The finding that guilt was the least represented emotion among the psychiatrists involved in cases of medical malpractice liability suggests that they did not feel responsible or at fault for their involvement.

This could be due to various factors, such as the belief that the patients’ conditions were beyond their control or that they had acted in accordance with the standard of care. However, it is worth noting that the absence of guilt does not necessarily imply a lack of empathy or concern for patients, as other emotions such as anxiety and anger may also reflect a sense of moral responsibility and care for patients’ health.

Cases of professional liability from negligence have been widely reported to have detrimental effects on the mental health of workers in a variety of specializations (29, 30), as well as well-known is the phenomenon of the ‘secondary victimization’ (31) and the occurrence of the so-called ‘Judicial Clinical Syndrome’ (32, 33).

A further element we investigated was whether there were differences between psychiatrists’ opinions based on the usual place of work. Psychiatrists working in settings that prioritize patient protection and care, such as hospital wards, residential and rehabilitation centers, and mental health service units in prisons, were more likely to face malpractice claims and to use DM more frequently, particularly when treating patients with suicidal or violent tendencies. This finding contrasts with general results that suggest a similar number of paid malpractice claims in inpatient and outpatient settings (34).

Possible explanations for our findings include the fact that psychiatrists in such settings may deal with more complex and high-risk patients who require coercive interventions, such as restraint or seclusion, which increase the risk of liability and complaints. Additionally, patients in these settings may have comorbid medical or social issues that require more extensive and coordinated care, increasing the potential for errors in treatment. Systemic and organizational challenges may also compromise the quality and safety of care in these environments, leading to a greater reliance on DM as a strategy to mitigate liability and protect providers. However, at least in the Italian context, this difference could be explained by the greater responsibility that psychiatrists because of PoG are obligated to shoulder in cases of patients’ self-harm or hetero-aggressive behaviors, as well as the possibility of suicide. It is also worth noting that our study focused on psychiatrists’ perceptions and experiences of professional liability and DM, rather than on objective measures of risk or liability, which may limit the generalizability and reliability of the findings.

The issue of PoG can be unsettling for both psychiatrists and patients, as it involves the re-emergence of outdated stigmas and uncertain custodial strategies. In our opinion, a possible dividing line to consider is the level of cognitive ability of the patient, a concept that is currently reflected in the clear hypotheses of involuntary treatments at a normative level. If the patient is competent and can provide valid consent, or if there are no requirements for involuntary treatment, the principle of freedom and autonomy must be respected by the psychiatrist, and a different PoG must be derived accordingly (35).

‘Open’ psychiatric services are generally characterized by a voluntary and long-lasting encounter between patient and psychiatrist, allowing for the creation of a therapeutic alliance that reduces the risk of malpractice claims (36). Our study found that psychiatrists in ‘closed’ environments sought contact with third parties (relatives, other professionals, support networks) more frequently, likely due to poor cohesion in the doctor-patient relationship.

Despite that, in ‘closed’ environments there is a greater liability for the protection of patients, making it easier to hold the psychiatrist accountable in cases of suicide or harmful behaviors. The inability to realize this obligation is one of the most common reasons for malpractice claims in psychiatric settings (37).

Our findings also suggest a significant relationship between awareness of practicing DM and evaluation of its use in the treatment of patients with suicidal tendencies. The positive correlation between all elements related to suicidal patients’ treatment and the use of DM suggests that Italian psychiatrists may be more inclined to use defensive practices in these cases.

The negative correlation between DM and medical-legal information suggests that healthcare providers may rely on defensive procedures to protect themselves against medical malpractice lawsuits when they lack access to medical-legal information. Inadequate knowledge of the law also emerged as a risk factor for DM in other medical contexts as well as its improvement was considered beneficial to increase clinicians’ confidence in their decision-making and to reassure them of the standards of the reasonableness of the law (38), Despite the multiple drivers of DM that exclude the possibility that education on the law alone would be sufficient to fix the problem, its amelioration could enhance the use of evidence-based guidelines and best practices by physicians only for clinical goals.

More than two-thirds of respondents indicated that they followed the guidelines and good clinical practices for both clinical and legal reasons. This highlights a tenacious fear of legal implications despite the application of evidence-based medicine. This has significant implications for the healthcare system, as DM, not evidence-based, can lead to unnecessary costs and procedures for patients, as well as an increase in medical errors. Conversely, the use of evidence-based guidelines and best practices can improve the quality of care and reduce the risk of adverse outcomes.

Law 24/2017 expressly provides for the use of clinical guidelines and good practices by medical professionals, both for the protection of patients’ healthcare and to avoid legal implications.

As for Law 24/2017, whose primary objective was to improve healthcare safety through the implementation of risk management, only 8 out of 177 respondents reported being satisfied with Italian legislation changes that occurred in 2017. This dissatisfaction cannot be due to psychiatrists’ lack of trust in risk management, given that the majority of psychiatrists believe that it reduces liability complaints.

Probably, the dissatisfaction may stem from the feeling that healthcare risks are primarily attributed to healthcare providers. It must be said that the WHO has recently emphasized that risk management should be an “integral part of strategic planning, implementation, and resource prioritization at all levels”; in other words, it should be the basis for an enabling ‘Risk Culture’, spread throughout institutions and not only among medical staffs (WHO, 2023) (39).

A further explanation could be represented in mental health by the need for guidelines and good practices adaptation to the specific case (‘the uniqueness of each patient’), with a personalization necessarily more stringent than for other medical fields (40). For this reason, when tailored care prevents compliance with guidelines indications, a risk for the psychiatrist is configured as it represents an element of negligence (deviation from the standard of care) (41).

Furthermore, although Law 24/2017 affirms the importance of clinical risk prevention by public and private health and social-health facilities, for some specific issues, such as suicide prevention, protocols are often generic, lacking risk stratification, specific indications for different risk degrees or diagnoses and standardized tools for risk assessment (7).

Another finding is the widespread opinion that training on clinical risk has not been sufficiently received, a condition that also occurs in other specializations (42). On this point, it should be emphasized that are mainly the younger psychiatrists who had less training and at the same time recognize performing more DM, despite admitting to having received fewer complaints than seniors.

The inverse correlation between age, seniority, and the perception of practicing defensive DM is consistent with similar data found by Reuveni et al. (11) for psychiatrists, as well as by other researchers in different medical specialties (43, 44). Several possible explanations for this correlation include differences in experience, training, the malpractice environment, technology, and personal factors. Senior psychiatrists may have more experience and confidence in their clinical decision-making, which could make them less likely to feel the need to practice defensively.

A myth that our study dispelled is that younger psychiatrists may have received more recent training that emphasizes the importance of risk management. As mentioned, it is above all senior psychiatrists who received more risk management training while a further critical figure emerged from the lack of relevance given to this matter in medical schools’ curricula, as occurred only in 18 cases out of 157.

Anyway, the malpractice environment also changed over time, with younger psychiatrists experiencing a more litigious context and maybe feeling more pressure to practice DM.

Finally, younger psychiatrists may simply be more risk-averse or, along with less seniority and practical experiences, have a different perception of the risks involved in medical practice compared to senior providers. This inference is corroborated by the fact that younger psychiatrists have a greater tendency to consult senior colleagues or send patients to other professionals.

However, further research is necessary to determine the specific reasons for the inverse correlation between age and the perception of practicing DM.

Particularly critical is the high frequency of DM among younger psychiatrists and professionals working in acute and high-intensity medical care facilities.

These findings, together with the issues related to the PoG, alarm about the possibility that young psychiatrists may avoid working in more problematic public services, with a risk to the sustainability of the National Health Service and adequate care of patients.

A possible cause for concern and confusion regarding PoG is given by judgments for infringement of patients’ rights (e.g., for excessive coercive treatments or use of pharmacological and/or physical restraints) or, conversely, for lack of surveillance and proactive actions, as well as for patients’ self-harm or other violent behaviors. It is, therefore, necessary to reaffirm what should be the elements to consider for the evaluation of negligence in malpractice trials: (i) analysis of malpractice cases based on the ‘reasonably-prudent-physician standard’ and not on a ‘standard of excellence’; (ii) clear evidence of a critical role of causation; (iii) foreseeability of the consequences of the acts or omissions, counteracting the danger of hindsight bias; (iv) consideration of plaintiffs’ role in bringing about their own injury or harm (comparative negligence) (41, 45).

The main limitations of the study are: (1) the small sample size: future research should replicate this study with a larger sample; (2) the scarcity of previous research on physicians’ malpractice concerns and coping strategies as there is a lack of established surveys or scales to measure these constructs; (3) the use of an *ad-hoc* survey for collecting self-report data which are subject to social desirability and recall biases. Minor limitations consist of a lack of questions about the proportion of malpractice complaints in terms of penal or civil matters and about the kind of most common types of malpractice complaints (e.g., diagnosis/treatment errors, poor communication, lack of informed consent, suicide/death). Further surveys including these issues may yield more insights into psychiatrists’ concerns.

## 5. Conclusion

Compared to the past, the cultural model of reference and the reconstruction of the psychiatrists’ tasks are different today. On the one hand, this allows for the rejection of residual custodial obligations, valuing the participatory role of psychiatric patients. On the other hand, it highlights the connection between the framework of the position of guarantee and allowed risk: the need to counter and mitigate a certain risk for the patient identifies and circumscribes, in the context of negligent liability, the precautionary rules. Thus, the link between the position of guarantee, preventive obligations, and precautionary rules is apparent. The rules of conduct with a precautionary content relevant to negligent imputation always presuppose and limit the duties of the physician, who cannot be asked to exercise more diligence, prudence, and expertise than what is required in the position of the guarantor. In this scenario, the role actually played by guidelines and accredited medical best practices within the scientific community must be investigated concerning self-injurious acts by psychiatric patients (primarily suicide), characterized by a physiologically unpredictable and uncontrollable risk.

In summary, this study provides valuable insights into the prevalence and impact of DM among psychiatrists in Italy, as well as the factors that contribute to its use, primarily PoG. The findings suggest that DM is a common phenomenon among Italian psychiatrists, with implications for almost all clinical situations investigated.

Moreover, our study underscores the need for research, training, and dialogue to improve the quality and safety of psychiatric care, reduce the use of DM, and promote access to mental health care.

In future perspectives, this research could provide useful insights for possible reviews of the laws and regulations governing clinical practice, in order to ensure the adoption of multidisciplinary protocols, the protection of patients’ and professionals’ rights, the development of guidelines and good practices by scientific societies, the promotion of the implementation of digital solutions such as telemedicine, in addition to the already mentioned need to improve training and enhance risk management culture.

The debate is animated by the tension between the need to protect patients’ rights and safety, and the risk of exposing psychiatrists to excessive litigation which could negatively impact clinical autonomy and therapeutic alliance. There is no easy solution, but addressing this issue also with evidence-based instruments is crucial to enable psychiatrists to practice in a supportive environment, build trust in the doctor-patient relationship and promote access to mental health care. Overall, this is a pivotal moment that could shape the development of psychiatry in Italy for years to come.

## Data availability statement

The original contributions presented in the study are included in the article/Supplementary material, further inquiries can be directed to the corresponding author.

## Author contributions

PSc, DM, and NDF: conceptualization. GD, VI, and SLP: data curation, in consultation with PF and VF. DM and NDF: formal analysis. PSc, PSa, VI, and GD: investigation. PSc and DM: methodology. DM, PSc, and NDF: writing-original draft. SLP, PF, and VF: writing-review and editing. PF and VF: supervision. All authors contributed to the article and approved the submitted version.

## In memoriam

This study is dedicated to the memory of the Italian psychiatrist Dr. Barbara Capovani (†2023) and to all healthcare professionals involved in cases of assaults at work.

## Conflict of interest

The authors declare that the research was conducted in the absence of any commercial or financial relationships that could be construed as a potential conflict of interest.

## Publisher’s note

All claims expressed in this article are solely those of the authors and do not necessarily represent those of their affiliated organizations, or those of the publisher, the editors and the reviewers. Any product that may be evaluated in this article, or claim that may be made by its manufacturer, is not guaranteed or endorsed by the publisher.
