# Supplementary material for: Vox clamantis in deserto: a survey among Italian psychiatrists on defensive medicine and professional liability
Source: Front Psychiatry. 2023 Aug 17;14:1244101. doi: 10.3389/fpsyt.2023.1244101 (PMC10469623; doi:10.3389/fpsyt.2023.1244101)
Supplement: Supplementary file 1 [file Data_Sheet_1.PDF]

Supplemental materials

Table S1. Summary of main findings

Sample characteristics:

- 254 psychiatrists
- 17.7% < 35 years old
- 53.1% between 36 and 50 years old
- 28.7% > 50 years old
- 60% women
- 90.9% specialists
- 75.2% consultants
- 68.9% worked in 'open' settings
- 30.7% worked in 'closed' settings

Defensive practice:

- 52% exposure to medical negligence risk
- 19.7% received an actual complaint
- 61% kept up with medico-legal literature but only 4.3-37.8% said it influenced their practice
- > 50% adopted defensive postures with suicidal patients (both hospitalization and medication)
- Lower tendency to hospitalize (13.8-51.6%) or refer (13.8-41.7%) violent patients but higher defensive behaviors otherwise
- 75.2% informed of severe side effects
- 31.1% informed of suicide risk with SSRIs
- 56.4-69.3% less likely to prescribe to pregnant or elderly patients
- 60.2% acknowledged practicing defensive medicine
- > 50% reported compromised the therapeutic relationship and goals of care
- 35.8% reported PoG influenced prescribing
- 18.1% reported PoG influenced involuntary hospitalization
- 11.9% reported non-clinical factors influenced hospitalization

Legislation and Risk Management:

- 68.9% reported concerns about liability laws
- 28.7-34.3% had not fully read Law 24/2017 or found improved protections (3.1%)
- Opinions on its application were heterogeneous
- 59.8% had risk management training, often in courses (44.6-35.7%)

Involved in legal proceedings:

- 50 (19.7%) had been involved in a complaint
- reported emotions were anxiety (86%), anger (82%), restlessness (62%) and loss of energy (58%)
- 40.9% reported impairment in functioning
- Guilt was least reported (18%)

T-Tests for Open-Closed Groups:

- Closed groups more likely to report involvement in malpractice (p=0.037) or complaints (p=0.031)
- Closed groups more likely to hospitalize (p=0.015) or contact supports (p<0.001) or consult experienced psychiatrists (p=0.024) for suicidal patients
- Closed groups more likely to contact supports (p=0.016) or consult experienced psychiatrists (p=0.015) or refer (p=0.029) for violent patients
- Open groups were more reluctant to prescribe to pregnant women (p=0.048)

ANOVAs:

- Older groups more likely to report liability or complaint involvement (p=0.015; p=0.005) or internal investigations (p<0.001)
- Younger groups more likely to consult senior psychiatrists (p<0.001) or refer (p=0.036) for suicidal patients
- Age inversely correlated with consulting senior psychiatrists (p<0.001); younger groups more likely to refer (p=0.038) or prescribe without cause (p=0.010) for violent patients
- Older groups more likely to inform of side effects (p<0.001), suicide risk (p=0.003) or risks (p<0.001) for elderly patients
- Younger/middle age groups more likely to acknowledge defensive practice (p=0.013)
- Age correlated with risk management training (p<0.001)

Correlations:

- Awareness of defensive practices correlated negatively with medico-legal knowledge (r=-0.126; p=0.045) but positively with influencing practice (r=0.189; p=0.009)
- All measures correlated positively with defensive practice except contacting supports for violent patients for suicidal/violent patients
- Informing patients/recording side effects did not correlate; lower doses for pregnant/elderly did (r=0.227; p<0.001)
- Perceiving goal of care as compromising care/relationships correlated with defensive practice (r=0.349; p<0.001) as did prioritizing it over clinical conditions (r=0.350; p<0.001) or involuntary hospitalization (r=0.346; p<0.001)
- External influences on hospitalization correlated with defensive practice (r=0.267; p<0.001)
- Younger age/less experience correlated negatively with defensive practice (r=-0.245; p<0.001)
- Involvement in legal issues did not correlate significantly

Table S2. Questions about feelings and functioning during the period of involvement in legal proceedings for professional liability

Questions	Total Answers	Answers
1. Have you been involved in a past civil or criminal complaint by one of your patients or their family members?	254/254	Yes n. (% 50)<br><br>No n. (% 204)
**If yes, during the period the complaint was filed, did you feel any of the following?**		
Anxious	50/50	Not at all n. 2 (% 4)<br><br>Slightly n. 5 (% 10)<br><br>To a certain extent n. 21 (% 42)<br><br>Very Much n. 22 (% 44)
Restless	50/50	Not at all n. 8 (% 16)<br><br>Slightly n. 11 (% 22)<br><br>To a certain extent n. 22 (% 44)<br><br>Very Much n. 9 (% 18)
Loss of energy / fatigue	50/50	Not at all n. 12 (% 24)<br><br>Slightly n. 9 (% 18)<br><br>To a certain extent n. 25 (% 50)<br><br>Very Much n. 4 (% 8)
Sleeping problems	50/50	Not at all n. 14 (% 28)<br><br>Slightly n. 9 (% 18)<br><br>To a certain extent n. 18 (% 36)<br><br>Very Much n.9 (% 18)
Anger	50/50	Not at all n. 4 (% 8)<br><br>Slightly n. 5 (% 10)<br><br>To a certain extent n. 13 (% 26)

		Very Much n. 28 (% 56)
Guilt	50/50	Not at all n. 30 (% 60)<br><br>Slightly n. 11 (% 22)<br><br>To a certain extent n. 7 (% 14)<br><br>Very Much n. 2 (% 4)
Distrustful	50/50	Not at all n. 8 (% 16)<br><br>Slightly n. 13 (% 26)<br><br>To a certain extent n. 20 (% 40)<br><br>Very Much n. 9 (% 18)
Impaired functioning in work, family relations, or social activities:	49/50	Not at all n. 16 (% 32,7)<br><br>Slightly n. 13 (% 26,5)<br><br>To a certain extent n.16 (% 32,7)<br><br>Very Much n. 4 (% 8,2)

Table S3. Comparison between psychiatrists working in 'open' and 'closed' contexts

	**'Open group' n.175 Mean (SD)**	**'Closed group' n.78 Mean (SD)**	**t**	**df**	**p**
Criminal Complaints					
**Being involved in a malpractice case (CriminalComplaints1)**	1,53 (0,501)	1,38 (0,490)	2,102	151,004	**0,037**
Being involved in an internal inquiry and/or convocated by a disciplinary board (CriminalComplaints2)	1,84 (0,368)	1,83 (0,375)	0,132	251	0,895
Education on medical liability (CriminalComplaints3)	1,41 (0,492)	1,36 (0,483)	0,701	251	0,484
Influence of education on medical liability in clinical pratctice (CriminalComplaints4)	2,41 (0,728)	2,57 (0,710)	-1,400	188	0,163
Suicidal Patients					
**Advises unwarranted hospitalisation (SuicidalPatients1)**	2,87 (1,102)	3,24 (1,107)	-2,458	251	**0,015**
Increases follow-up (SuicidalPatients2)	3,89 (1,025)	3,95 (0,966)	-0,418	251	0,677
**Initiates contact with family (SuicidalPatients3)**	3,60 (1,077)	4,12 (0,806)	-4,215	194,142	**0,000**
**Consults senior psychiatrist (SuicidalPatients4)**	2,85 (1,099)	3,19 (1,117)	-2,267	251	**0,024**
Refers to another professional (SuicidalPatients5)	2,72 (1,029)	2,95 (1,169)	-1,563	249	0,119
Prescribes medication without indication (SuicidalPatients6)	2,63 (1,021)	2,83 (1,174)	-1,287	129,044	0,200
Violent Patients					
**Violent Patients**					

Advises unwarranted hospitalisation (ViolentPatients1)	2,32 (0,897)	2,56 (1,118)	-1,652	120,941	0,101
Increases follow-up (ViolentPatients2)	3,11 (1,098)	3,17 (1,185)	-0,354	250	0,723
**Initiates contact with family (ViolentPatients3)**	3,35 (1,067)	3,71 (1,122)	-2,429	250	**0,016**
**Consults senior psychiatrist (ViolentPatients4)**	3,01 (1,096)	3,37 (1,106)	-2,447	251	**0,015**
**Refers to another professional (ViolentPatients5)**	2,45 (0,979)	2,80 (1,222)	-2,216	119,105	**0,029**
Prescribes medication without indication (ViolentPatients6)	3,06 (1,089)	3,31 (1,103)	-1,664	250	0,097
Patients Medication					
Informs about severe yet rare side effects (PatientsMedication1)	3,75 (1,243)	3,69 (1,262)	0,331	251	0,741
Records that explained about side effects (PatientsMedication2)	2,58 (1,479)	2,76 (1,452)	-0,867	251	0,387
Informs of increased risk of suicidality (PatientsMedication3)	2,21 (1,283)	2,21 (1,283)	-1,811	128,307	0,073
Pregnant patients					
**Avoids medication altogether (Pregnant1)**	3,06 (1,179)	2,74 (1,178)	1,991	250	**0,048**
Collects different consent (Pregnant2)	3,40 (1,706)	3,42 (1,725)	-0,114	250	0,910
Prescribes a smaller dosage (Pregnant3)	3,47 (1,376)	3,44 (1,401)	0,188	250	0,851
Elderly patients					
Informs of cerebrovascular diseases risk (Elderly1)	3,44 (1,401)	3,00 (1,441)	0,678	250	0,498

Prescribes a smaller dosage (Elderly2)	4,22 (0,852)	4,18 (0,936)	0,325	250	0,746
Defensive Medicine					
Admission of practising defensive medicine (DefensiveMedicine1)	2,92 (1,053)	3,08 (1,171)	-1,057	251	0,291
Believe the position of guarantee influences physicians' relationships with certain types of patients, e.g., those with violent behavior, suicidal ideation, dual diagnoses, criminal convictions (DefensiveMedicine2)	2,90 (1,145)	2,82 (1,254)	0,490	250	0,625
Believe the position of guarantee adversely affects the clinical outcome of certain types of patients, e.g., those with violent behavior, suicidal ideation, dual diagnoses, criminal convictions (DefensiveMedicine3)	2,92 (1,177)	2,70 (1,278)	1,324	250	0,187
Prioritize the position of guarantee over patient needs in prescribing drugs (DefensiveMedicine4)	2,41 (0,995)	2,25 (1,041)	1,152	250	0,250
Prioritize the position of guarantee over patient needs in involuntary hospitalization (DefensiveMedicine5)	1,91 (0,899)	1,84 (0,961)	0,554	249	0,580
Involuntarily hospitalization due to external pressure (rather than a medical need) (DefensiveMedicine6)	1,77 (0,793)	1,78 (0,754)	-0,126	250	0,900
'Gelli-Bianco' Law 24/2017					
Kind of legal area at greatest risk for own job (GelliBianco1)	2,58 (0,689)	2,58 (0,676)	-0,017	250	0,987

Education on law 24/2017- so-called Gelli–Bianco law (GelliBianco2)	1,99 (0,851)	1,96 (0,834)	0,238	250	0,812
Opinion on increased guarantees for psychiatrists provided by Law 24/2017 (GelliBianco3)	2,56 (0,559)	2,54 (0,646)	0,241	174	0,810
Legal area where Law 24/2017 increased guarantees for psychiatrists (GelliBianco4)	1,98 (0,864)	2,25 (0,838)	-1,879	166	0,062
Participation in training on clinical risk (GelliBianco5)	1,41 (0,492)	1,40 (0,493)	0,123	251	0,902
Adequacy of clinical risk management training (GelliBianco7)	2,28 (0,643)	2,26 (0,661)	0,178	246	0,859
Opinion on the utility of risk management in reducing medical claims (GelliBianco8)	1,11 (0,311)	1,12 (0,326)	-0,221	187	0,825
Reasons for adhering to guidelines (GelliBianco9)	1,93 (0,565)	1,96 (0,502)	-0,393	248	0,695
Demographic					
Age (years)	46,12 (10,996)	43,95 (10,621)	1,465	250	0,144
Age by groups*	2,138 (0,657)	2,038 (0,711)	1,083	250	0,280
Seniority (years)	14,92 (10,814)	12,26 (9,944)	1,769	236	0,078
Others					
**Involvement in complaints**	1,85 (0,362)	1,73 (0,446)	2,165	251	**0,031**
Acknowledgement of defensive practice	1,600 (0,491)	1,615 (0,489)	-0,230	251	0,818

- * Groups: 1= < 35; 2 = 36-50; 3 = > 50
- Bold values denote statistical significance at the p < 0.05 level

Table S4. Analysis of variance of the three groups of participants divided according to different age

	**'<35 group' n. 46 Mean (SD)**	**'36-50 group' ' n. 135 Mean (SD)**	**'>50 group'' n. 73 Mean (SD)**	**F**	**p**	**Post hoc**
Criminal Complaints						
**Being involved in a malpractice case (CriminalComplaints1)**	1,67 (0,477)	1,47 (0,501)	1,40 (0,493)	*4,489	**0,015**	**a>b, a>c**
**Being involved in an internal inquiry and/or convocated by a disciplinary board (CriminalComplaints2)**	1,98 (0,149)	1,88 (0,324)	1,68 (0,468)	*13,469	**<0,001**	**a>b>c**
Education on medical liability (CriminalComplaints3)	1,51 (0,506)	1,39 (0,488)	1,32 (0,468)	*2,202	0,105	
Influence of education on medical liability in clinical pratctice (CriminalComplaints4)	2,41 (0,743)	2,44 (0,703)	2,49 (0,735)	0,143	0,867	
Suicidal Patients						
Advises unwarranted hospitalisation (SuicidalPatients1)	2,91 (1,041)	3,07 (1,111)	2,86 (1,170)	0,964	0,383	
Increases follow-up (SuicidalPatients2)	3,96 (1,021)	3,98 (0,973)	3,74 (1,068)	1,385	0,252	
Initiates contact with family (SuicidalPatients3)	3,73 (1,074)	3,72 (1,034)	3,85 (0,995)	0,399	0,672	
**Consults senior psychiatrist (SuicidalPatients4)**	3,56 (0,990)	2,95 (1,039)	2,58 (1,166)	11,708	**<0,001**	**a>b, a>c**
**Refers to another professional (SuicidalPatients5)**	3,02 (1,045)	2,85 (1,022)	2,53 (1,156)	3,363	**0,036**	**a>c**

Prescribes medication without indication (SuicidalPatients6)	2,89 (1,166)	2,72 (1,090)	2,54 (0,963)	1,477	0,230	
Violent Patients						
Advises unwarranted hospitalisation (ViolentPatients1)	2,39 (0,841)	2,32 (0,928)	2,53 (1,119)	*0,985	0,314	
Increases follow-up (ViolentPatients2)	3,11 (1,125)	3,09 (1,109)	3,25 (1,152)	0,479	0,620	
Initiates contact with family (ViolentPatients3)	3,43 (1,043)	3,38 (1,092)	3,64 (1,123)	1,428	0,242	
**Consults senior psychiatrist (ViolentPatients4)**	3,71 (0,895)	3,16 (1,045)	2,66 (1,157)	*15,291	**<0,001**	**a>b>c**
**Refers to another professional (ViolentPatients5)**	2,93 (1,043)	2,50 (1,074)	2,45 (1,039)	3,305	**0,038**	**a>b, a>c**
**Prescribes medication without indication (ViolentPatients6)**	3,59 (0,871)	3,06 (1,084)	3,01 (1,184)	*6,411	**0,010**	**a>b, a>c**
Patients Medication						
**Informs about severe yet rare side effects (PatientsMedication1)**	3,38 (1,248)	3,59 (1,254)	4,23 (1,100)	9,166	**<0,001**	**a<c, b<c**
Records that explained about side effects (PatientsMedication2)	2,56 (1,391)	2,50 (1,403)	2,97 (1,624)	*2,218	0,082	
**Informs of increased risk of suicidality (PatientsMedication3)**	2,18 (1,386)	2,13 (1,212)	2,78 (1,521)	*5,126	**0,003**	**b<c**
Pregnant patients						
Avoids medication altogether (Pregnant1)	2,67 (1,000)	2,99 (1,179)	3,10 (1,282)	1,920	0,149	
Collects different consent (Pregnant2)	3,36 (1,721)	3,46 (1,702)	3,34 (1,734)	0,125	0,883	

Prescribes a smaller dosage (Pregnant3)	3,56 (1,216)	3,51 (1,386)	3,30 (1,469)	*0,659	0,501	
Elderly patients						
**Informs of cerebrovascular diseases risk (Elderly1)**	2,80 (1,408)	2,84 (1,383)	3,77 (1,318)	12,244	**<0,001**	**a<c, b<c**
Prescribes a smaller dosage (Elderly2)	4,13 (0,842)	4,28 (0,826)	4,12 (0,985)	0,906	0,406	
Defensive Medicine						
Admission of practising defensive medicine (DefensiveMedicine1)	3,20 (1,100)	3,00 (1,015)	2,73 (1,170)	2,934	0,055	
Believe the position of guarantee influences physicians' relationships with certain types of patients, e.g., those with violent behavior, suicidal ideation, dual diagnoses, criminal convictions (DefensiveMedicine2)	2,95 (1,160)	2,96 (1,145)	2,66 (1,216)	1,683	0,188	
Believe the position of guarantee adversely affects the clinical outcome of certain types of patients, e.g., those with violent behavior, suicidal ideation, dual diagnoses, criminal convictions (DefensiveMedicine3)	3,07 (1,208)	2,92 (1,159)	2,59 (1,256)	2,688	0,070	
Prioritize the position of guarantee over patient needs in prescribing drugs (DefensiveMedicine4)	2,61 (1,017)	2,34 (0,948)	2,23 (1,100)	2,007	0,137	
Prioritize the position of guarantee over patient needs in involuntary hospitalization (DefensiveMedicine5)	1,84 (0,963)	1,98 (0,880)	1,75 (0,954)	1,485	0,228	

Involuntarily hospitalization due to external pressure (rather than a medical need) (DefensiveMedicine6)	1,73 (0,758)	1,82 (0,790)	1,68 (0,780)	0,795	0,453	
‘Gelli-Bianco’ - Law 24/2017						
Kind of legal area at greatest risk for own job (GelliBianco1)	2,43 (0,695)	2,59 (0,672)	2,63 (0,717)	1,226	0,295	
Education on law 24/2017- so-called Gelli–Bianco law (GelliBianco2)	2,56 (0,698)	2,59 (0,538)	2,49 (0,601)	0,346	0,708	
Opinion on increased guarantees for psychiatrists provided by Law 24/2017 (GelliBianco3)	2,56 (0,698)	2,59 (0,538)	2,49 (0,601)	0,472	0,625	
Legal area where Law 24/2017 increased guarantees for psychiatrists (GelliBianco4)	2,04 (0,841)	2,11 (0,862)	2,00 (0,886)	0,472	0,625	
**Participation in training on clinical risk (GelliBianco5)**	1,69 (0,468)	1,41 (0,493)	1,22 (0,417)	*15,303	**<0,001**	**a>b>c**
Adequacy of clinical risk management training (GelliBianco7)	2,23 (0,649)	2,32 (0,608)	2,22 (0,716)	0,594	0,553	
Opinion on the utility of risk management in reducing medical claims (GelliBianco8)	1,06 (0,250)	1,12 (0,321)	1,13 (0,339)	0,439	0,645	
Reasons for adhering to guidelines (GelliBianco9)	1,91 (0,426)	1,97 (0,519)	1,89 (0,636)	*0,572	0,558	
Others						
**Involvement in complaints**	1,96 (0,208)	1,81 (0,396)	1,71 (0,456)	*9,705	**0,005**	**a>b, a>c**

**Acknowledgement of defensive practice**	1,7111 (0,458)	1,6370 (0,482)	1,4658 (0,502)	*4,283	**0,013**	**a>c, b>c**

- *Levene's test significant
- Bold values denote statistical significance at the $p < 0.05$ level
- Post hoc: Games-Howell
